# Supplementary material for: Odor-regulated oviposition behavior in an ecological specialist
Source: Nat Commun. 2023 May 26;14:3041. doi: 10.1038/s41467-023-38722-z (PMC10219952; doi:10.1038/s41467-023-38722-z)
Supplement: Supplementary file 1 — Supplementary Information [file 41467_2023_38722_MOESM1_ESM.pdf]

Supplementary Information

Odor-regulated oviposition behavior in an ecological specialist

Raquel Álvarez-Ocaña, Michael P. Shahandeh, Vijayaditya Ray,
Thomas O. Auer, Nicolas Gompel and Richard Benton

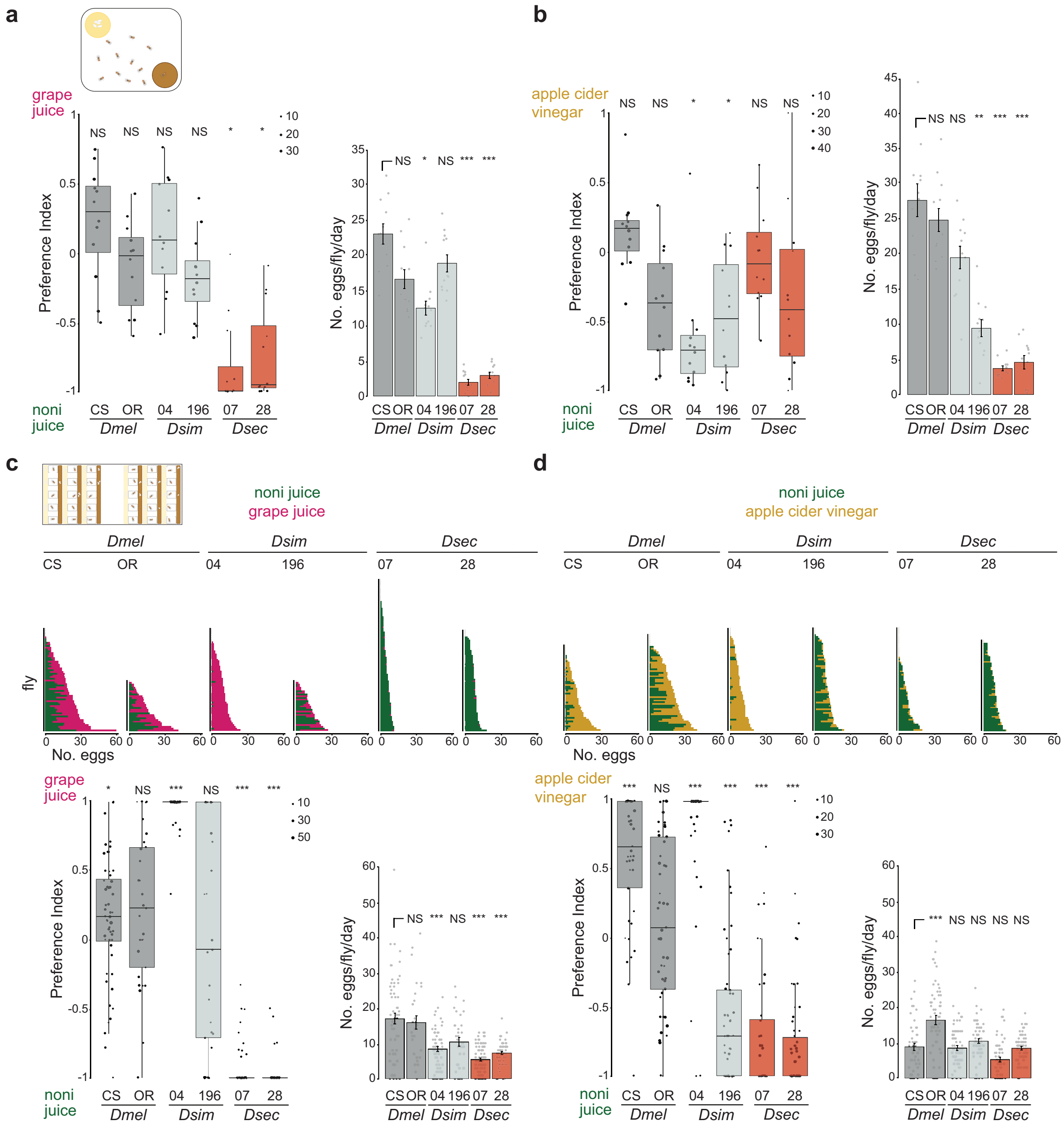

Supplementary Figure 1. Oviposition preference assays using instant medium-based substrates.

a Group oviposition preference assays for noni juice versus grape juice in instant medium using the same strains as in Fig. 1c. Left: box plots of oviposition preference index. Statistically-significant differences from 0 (no preference) are indicated (as in Fig. 1c); *N* = 12 (representing 4 assays of 40-80 flies, each scored on 3 successive days with fresh oviposition plates each day). Right: bar plots of egg-laying rate. Mean values ± standard error of the mean (SEM) are shown in these and all other bar plots. Statistically-significant differences from the *D. melanogaster* Canton-S (CS) strain are indicated (as in Fig. 1c).

b Group oviposition preference assay, as in **a**, for noni juice versus apple cider vinegar; *N* = 12 (representing 4 assays of 40-80 flies, each scored on 3 successive days with fresh oviposition plates each day).

c Single-fly oviposition preference assay for noni juice versus grape juice in instant medium for the same strains as in **a**. Top: total number of eggs laid in each

substrate by each female. Bottom left: oviposition preference index. Bottom right: egg-laying rate.

d Single-fly oviposition preference assays, as in **c**, for noni juice versus apple cider vinegar; *N* = 43-60 flies across 2 technical replicates.

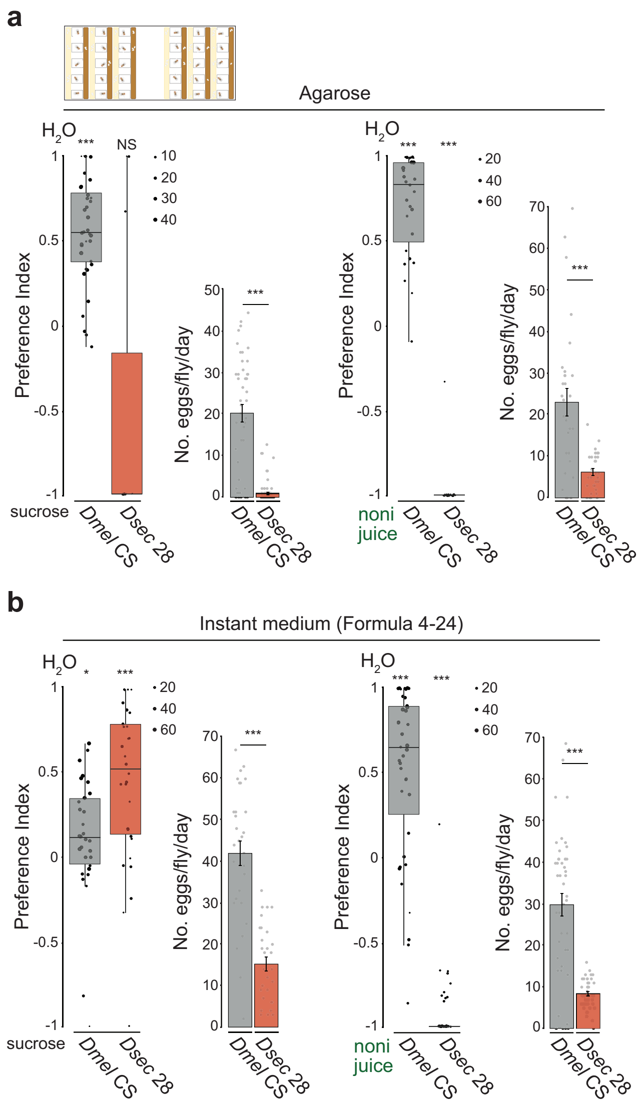

Supplementary Figure 2. Establishment of single-fly oviposition assays.
a Single-fly oviposition preference assays testing H_2O versus either 150 mM sucrose or noni juice in agarose. For the box-plots of oviposition preference index, statistically-significant differences from 0 (no preference) are indicated: *** $P < 0.001$; * $P < 0.05$; NS (not significant) $P > 0.05$ (Wilcoxon test with Bonferroni correction for multiple comparisons); N = 30-45 flies across 1-2 technical replicates. Preference of *D. melanogaster* for the plain substrate resembles previous observations[1]. Bar plots of egg-laying rate indicate mean values ± SEM. Statistical comparisons between genotypes are shown: *** $P < 0.001$ (two-sample t-test).
b Single-fly oviposition preference assays, as in **a**, using instant medium instead of agarose; N = 30-45 flies across 1-2 technical replicates. The higher egg-laying rate of both species on instant medium compared to agarose substrates might reflect differences in chemical composition, texture and/or moistness. Most experiments were performed using simpler agarose substrates, to minimize interference with responses to complex stimuli (i.e., juices). However, instant medium substrates were used in most single-odor experiments, as egg-laying rate was very low on agarose substrates with single chemical stimuli, especially for *D. sechellia* strains.

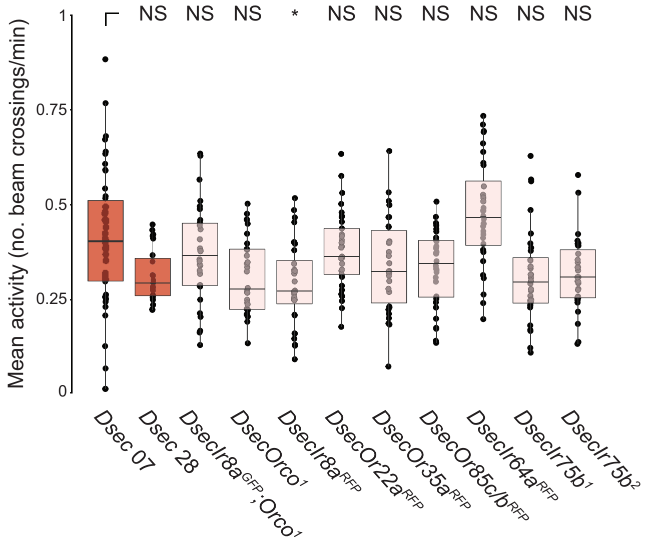

Supplementary Figure 3. Locomotor activity of olfactory receptor mutants. Mean activity of the indicated *D. sechellia* strains represented by the number of beam crossings per minute per fly recorded over 3 complete 12 h light: 12 h dark cycles. Statistically-significant differences from *Dsec* 07 (the genetic background strain for all mutants) are indicated: * $p < 0.05$; NS $P > 0.05$ (Wilcoxon test with Bonferroni correction for multiple comparisons); N = 23-46 flies per genotype across at least 2 technical replicates.

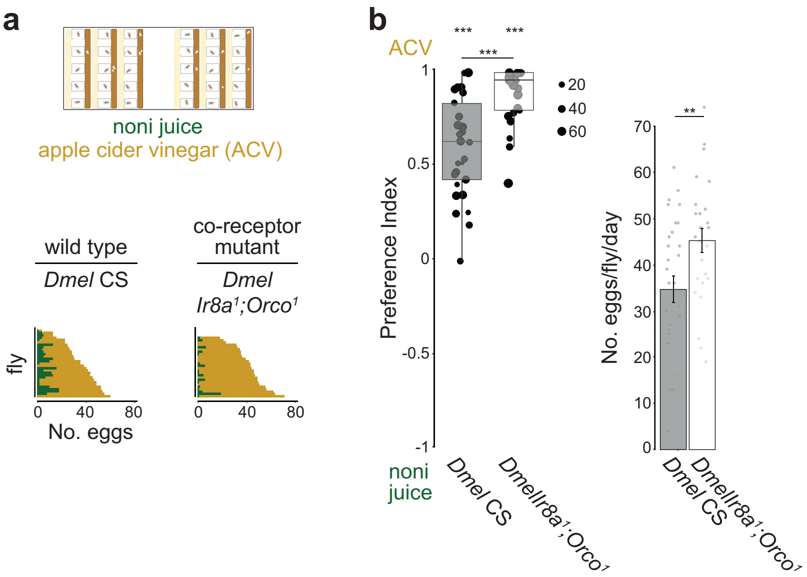

Supplementary Figure 4. Near-anosmic *D. melanogaster* do not display defects in oviposition behavior.

a Single-fly oviposition preference assays for apple cider vinegar versus noni juice in agarose for the wild-type *D. melanogaster* Canton-S (CS) and near-anosmic *DmelIr8a^1;Orco^1* mutants. The plots show the number of eggs laid per fly; *N* = 26-29 flies across 1 technical replicate.

b Left: oviposition preference index for the assays shown in **a**. Statistically-significant differences from 0 (no preference) are indicated: *** $P < 0.001$ (Wilcoxon test with Bonferroni correction for multiple comparisons). The two genotypes are statistically-significantly different: $P = 6.334\times10^{-5}$ (Wilcoxon test with Bonferroni correction). Right: egg-laying rate. The two strains are statistically-significantly different: ** $P < 0.01$ (Kruskal-Wallis rank sum test with Nemenyi post-hoc test). Note, however, that as the *Ir8a^1;Orco^1* mutant is not in the CS genetic background, it is unclear if the slight increase in preference for apple cider vinegar and in egg-laying rate in the mutant strain reflects a function for these olfactory co-receptors or an effect of genetic background.

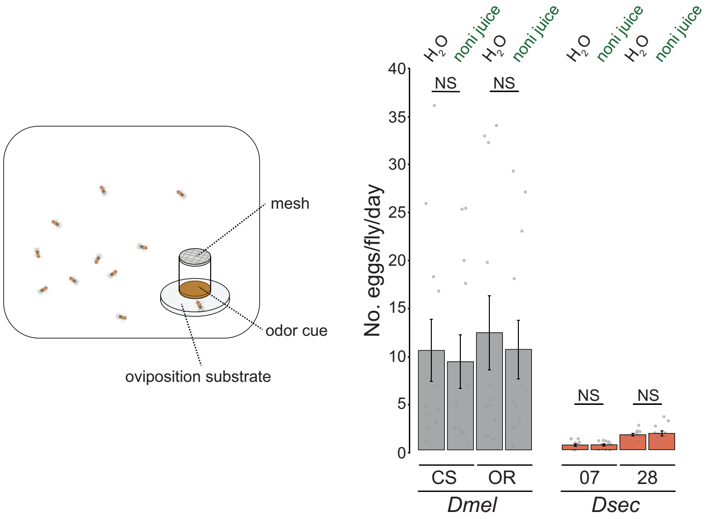

Supplementary Figure 5. Noni odor cues are not sufficient to promote egg-laying behavior.

Left: schematic of the one-choice group oviposition assay in the presence of a non-accessible noni juice source or H_2O control. Agarose plates supplemented with 150 mM sucrose were provided as oviposition substrates. Right: egg-laying rate. No statistically-significant differences between the conditions were observed: NS $P > 0.05$ (Kruskal-Wallis rank sum test with Nemenyi post-hoc test); $N = 12$ (representing 4 assays, each scored on 3 successive days with fresh oviposition plates each day).

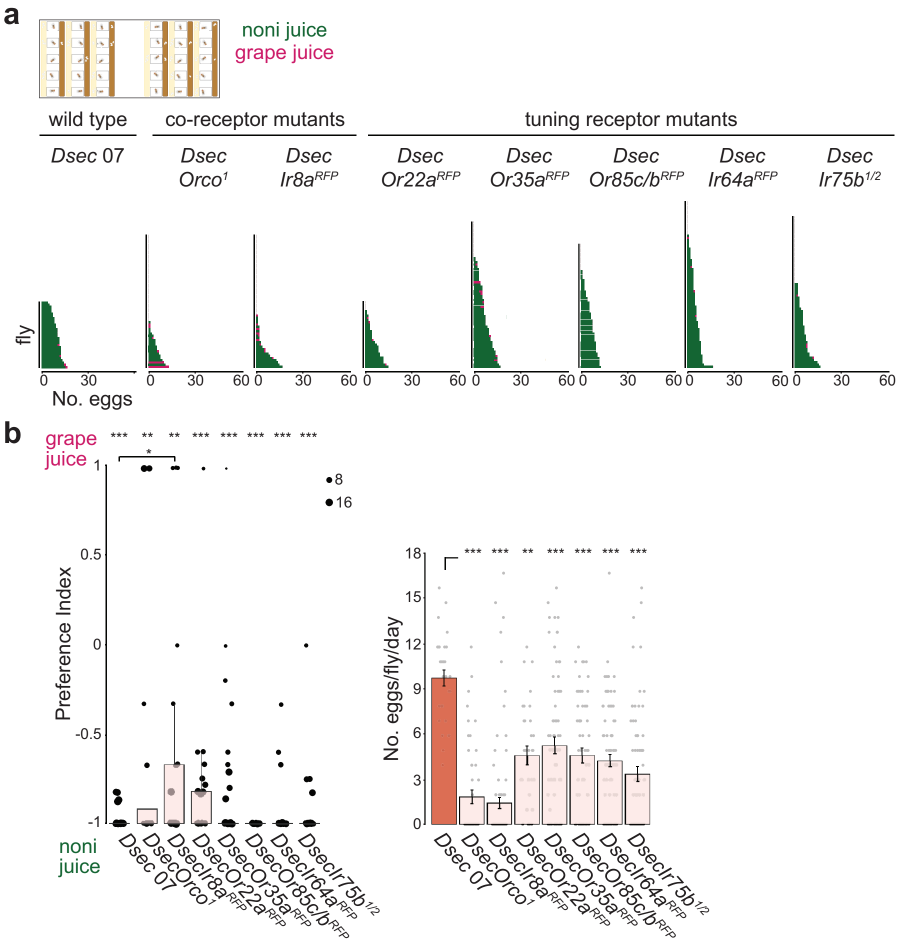

Supplementary Figure 6. Olfactory pathways required for *D. sechellia* oviposition.

a Single-fly oviposition preference assays for noni juice versus grape juice in agarose for the indicated genotypes. The plots show the number of eggs laid per fly; N = 30-60 flies across 1-2 technical replicates.

b Single-fly oviposition assay for the same strains and conditions as in **a**. Left: oviposition preference index. Statistically-significant differences from 0 (no preference) are indicated: *** $P < 0.001$; ** $P < 0.01$; * $P < 0.05$ (Wilcoxon test with Bonferroni correction for multiple comparisons); N = 30-60 flies, 1-2 assays. *Dsec 07* and *DsecIr8a^{RFP}* show a statistical difference: $P = 0.0223$ (Wilcoxon test with Bonferroni correction). Right: egg-laying rate in these assays. *** $P < 0.001$; ** $P < 0.01$ (Kruskal-Wallis rank sum test with Nemenyi post-hoc test).

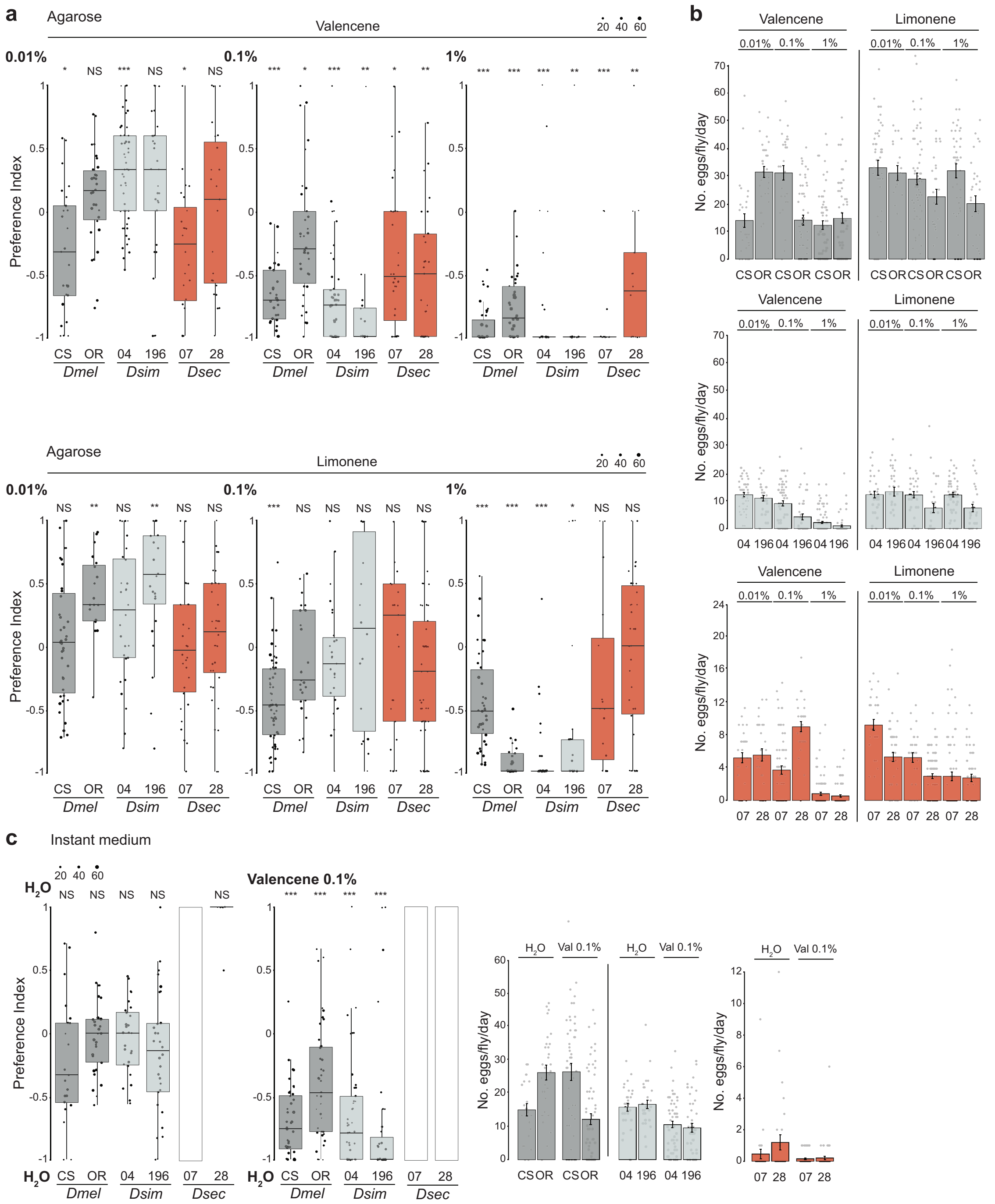

Supplementary Figure 7. Influence of valencene and limonene on oviposition behavior.

a Single-fly oviposition assays for valencene and limonene at the indicated concentrations. Odors were diluted in apple cider vinegar (for *D. melanogaster* and *D. simulans*) or noni juice (for *D. sechellia*) and agarose. For further information about fly strains and statistical analyses, see the legend to Fig. 1c. Statistically-significant differences from 0 (no preference) are indicated: *** $P < 0.001$; ** $P < 0.01$; * $P < 0.05$; NS $P > 0.05$ (Wilcoxon test with Bonferroni correction for multiple comparisons); N = 30-90 flies across 1-3 technical replicates.

b Bar plots of egg-laying rates for flies in the assays in **a**.

c Single-fly oviposition assays of the same strains as in **a**, with an instant medium substrate. Left: oviposition preference index in control (H_2O versus H_2O) or

experimental (0.1% valencene versus H_2O) conditions. Statistically-significant differences from 0 (no preference) are indicated: *** P < 0.001; NS P > 0.05 (Wilcoxon test with Bonferroni correction for multiple comparisons); N = 30-60 flies across 1-2 technical replicates. Right: egg-laying rate in these assays (Val = valencene). For some strains (indicated with a white rectangle), the low number of flies laying eggs prevented calculation of a preference index.

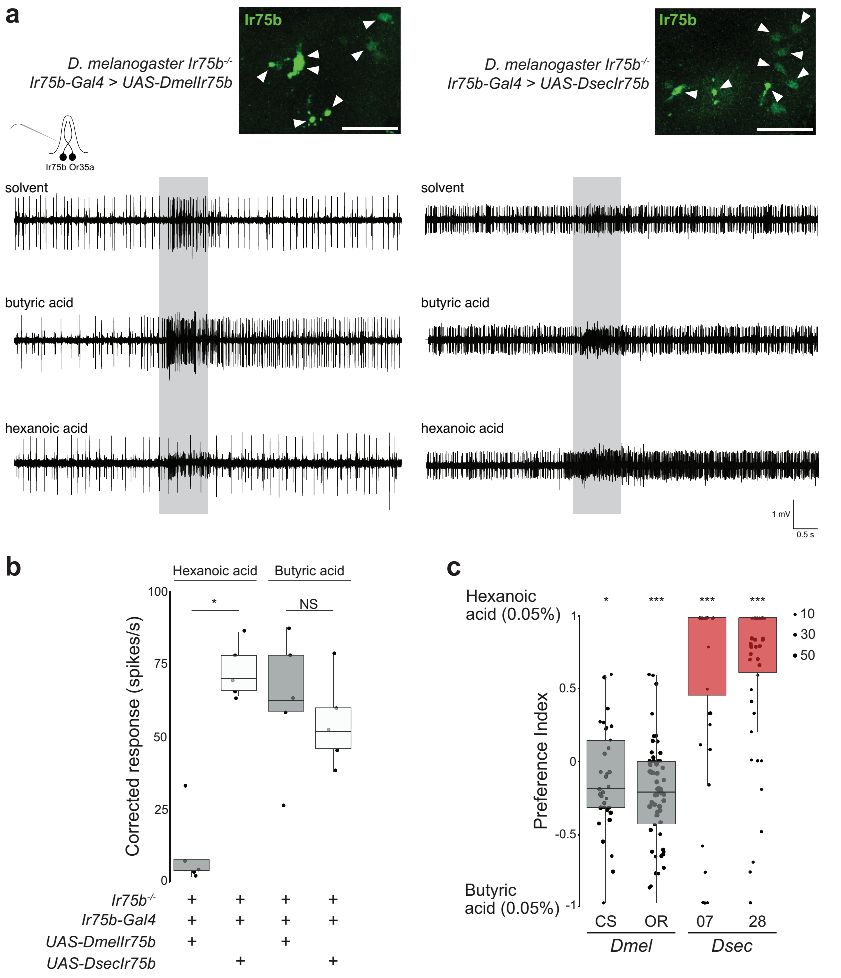

Supplementary Figure 8. Validation of the expression and species-specific function of heterologously-expressed Ir75b variants.

a Top: immunohistochemistry for Ir75b on whole-mount antennae of flies expressing *D. melanogaster* or *D. sechellia* Ir75b in *D. melanogaster Ir75b* mutant neurons (see Fig. 6d for the full genotypes). Scale bars, 25 μm. Although immunofluorescence signals of soma (arrowheads) were variable between neurons within an antenna – possibly reflecting the heterogeneous strength of the *Ir75b* promoter-Gal4 driver – they were comparable between the two genotypes. 5 and 6 antennae were examined for the *D. melanogaster* and *D. sechellia* Ir75b rescue genotypes, respectively. Bottom: representative traces of extracellular recordings from the same genotypes of neuronal responses in antennal sensilla housing Ir75b neurons (and Or35a neurons) to the indicated odors or solvent (H_2O). The grey bars indicate the stimulus period (1 s).

b Quantification of odor-evoked electrophysiological responses of the indicated genotypes (see the legend to Fig. 6d for the full genotypes). Each dot represents a measurement from an independent olfactory sensillum. Statistically-significant differences are indicated. * $P < 0.05$; NS $P > 0.05$ (pairwise Wilcoxon test with Bonferroni correction).

c Single-fly oviposition preference assays for hexanoic acid versus butyric acid in instant medium for the indicated wild-type strains of *D. melanogaster* and *D. sechellia*. Statistically-significant differences from 0 (no preference) are indicated. *** $P < 0.001$; * $P < 0.05$ (Wilcoxon test with Bonferroni correction for multiple comparisons); N = 34-60 flies across 2 technical replicates.

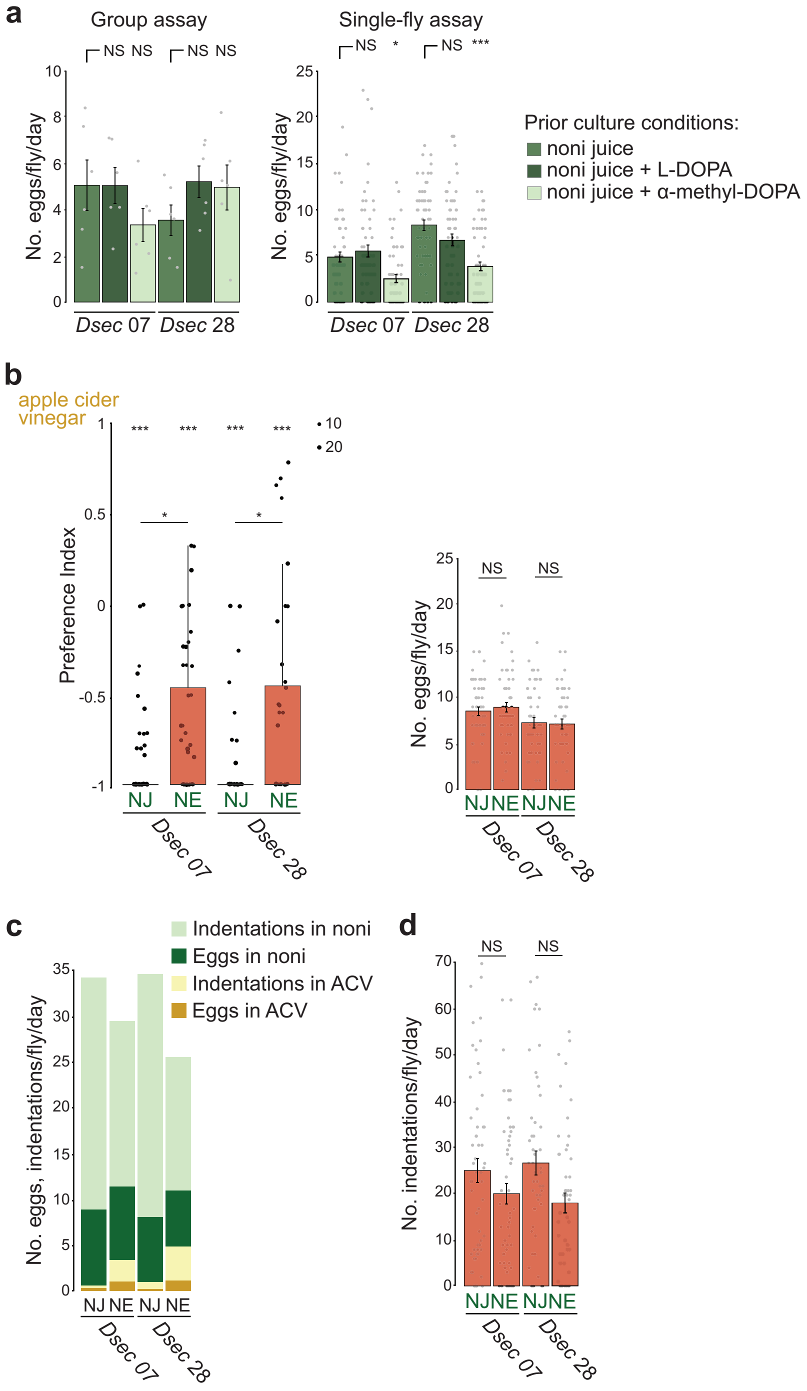

Supplementary Figure 9. Impact of modulation of L-DOPA levels, and of fresh noni extract, on oviposition behavior of *D. sechellia*.

a Egg-laying rate of flies that had been grown on control standard noni juice food or that supplemented with L-DOPA or α-methyl-DOPA (see Methods). Left: group oviposition assays, representing 2 assays of either 10 (*D. melanogaster*) or 20 (*D. sechellia*) flies each, scored on 3 successive days with a fresh non-supplemented noni paste oviposition plate provided each day. Right: single-fly oviposition assays using noni paste as oviposition substrate on both sides of the chamber. Statistically-significant differences from flies grown on non-supplemented noni juice food are shown: *** $P < 0.001$; * $P < 0.05$; NS $P > 0.05$ (Kruskal-Wallis rank sum

test with Nemenyi post-hoc test); *N* = 60 flies across 2 technical replicates.
b Single-fly oviposition preference assays for commercial noni juice (NJ) or fresh noni extract (NE) versus H_2O in agarose for the indicated wild-type strains of *D. sechellia*. Left: oviposition preference index. Statistically-significant differences from 0 (no preference) are indicated. *** $P < 0.001$ (Wilcoxon test with Bonferroni correction for multiple comparisons). Statistically-significant differences exist between experimental conditions (NJ or NE) in both *Dsec 07* and *Dsec 28*: * $P < 0.05$ (pairwise Wilcoxon test with Bonferroni correction); *N* = 49-56 flies across 2 technical replicates. Right: egg-laying rate. NS $P > 0.05$ (Kruskal-Wallis rank sum test with Nemenyi post-hoc test).
c Rate and distribution of egg-laying and indentation formation of the flies in the assays in **b**.
d Rate of indentation formation observed in the experiments in **b**. No statistically-significant differences were observed between conditions: NS $P > 0.05$ (Kruskal-Wallis rank sum test with Nemenyi post-hoc test).

Supplementary Table 1. *Drosophila* strains.

Strain	Source	Identifier
D. melanogaster wildtype Canton-S (CS)		
D. melanogaster wildtype Oregon-R (OR)		
D. melanogaster ovo^{D1} *v24 / Y / C(1)Dx, y f*	Daniel Pauli	
D. melanogaster $Ir8a^{1}$	[2]	RRID:BDSC_41744
D. melanogaster $Orco^{1}$	[3]	RRID:BDSC_23130
D. melanogaster $Ir75b^{DsRed}$	[4]	
D. melanogaster Ir75b-Gal4	[5]	
D. melanogaster UAS-DmelIr75b	[5]	
D. melanogaster UAS-DsecIr75b	[5]	
D. simulans wildtype 04	*Drosophila* Species Stock Center [DSSC]	14021-0251.004
D. simulans wildtype 196	DSSC	14021-0251.196
D. sechellia wildtype 07	DSSC	14021-0248.07
D. sechellia wildtype 28	DSSC	14021-0248.28
D. sechellia $Orco^{1}$ (strong hypomorph)	[6]	
D. sechellia $Or22a^{RFP}$	[6]	
D. sechellia $Or85c/b^{RFP}$	[6]	
D. sechellia $Or35a^{RFP}$	[6]	
D. sechellia $Ir8a^{GFP}$	[6]	
D. sechellia $Ir8a^{RFP}$	[6]	
D. sechellia $Ir64a^{RFP}$	[6]	
D. sechellia $Ir75b^{1}$	[6]	
D. sechellia $Ir75b^{2}$	[6]	

Supplementary Table 2. Chemicals.

Chemical	Supplier	CAS
Agarose	Promega	-
Apple cider vinegar	Migros	-
BACTO Agar	BD	214010
Balsamic vinegar	Antica Modena	-
Butyric acid	Sigma-Aldrich	107-92-6
3,4-dihydroxyphenylalanine (L-DOPA)	Sigma-Aldrich	53587-29-4
Formula 4-24® instant *Drosophila* medium (“instant medium”)	Carolina Biological Supply Company	-
Grape juice	Beutelsbacher Bio	-
2-heptanone	Sigma-Aldrich	110-43-0
Hexanoic acid	Sigma-Aldrich	142-62-1
Limonene	Sigma-Aldrich	5989-27-5
α-methyl-DOPA	Sigma-Aldrich	41372-08-1
Methyl hexanoate	Sigma-Aldrich	106-70-7
Noni juice	Raab Vitalfood Bio	-
Octanoic acid	Sigma-Aldrich	124-07-2
Sucrose	Sigma-Aldrich	57-50-1
Valencene	Sigma-Aldrich	4630-07-3

Supplementary References

1. Gou B, Zhu E, He R, Stern U, Yang CH. High Throughput Assay to Examine Egg-Laying Preferences of Individual *Drosophila melanogaster*. *J Vis Exp.* e53716 (2016).

2. Abuin L, Bargeton B, Ulbrich MH, Isacoff EY, Kellenberger S, Benton R. Functional architecture of olfactory ionotropic glutamate receptors. *Neuron.* **69**, 44-60 (2011).

3. Larsson MC, Domingos AI, Jones WD, Chiappe ME, Amrein H, Vosshall LB. *Or83b* encodes a broadly expressed odorant receptor essential for *Drosophila* olfaction. *Neuron.* **43**, 703-714 (2004).

4. Mika K*, et al.* Olfactory receptor-dependent receptor repression in *Drosophila*. *Science Advances.* **7**, eabe3745 (2021).

5. Prieto-Godino LL*, et al.* Evolution of Acid-Sensing Olfactory Circuits in Drosophilids. *Neuron.* **93**, 661-676 e666 (2017).

6. Auer TO*, et al.* Olfactory receptor and circuit evolution promote host specialization. *Nature.* **579**, 402-408 (2020).
