# Supplementary material for: Odor-regulated oviposition behavior in an ecological specialist
Source: Nat Commun. 2023 May 26;14:3041. doi: 10.1038/s41467-023-38722-z (PMC10219952; doi:10.1038/s41467-023-38722-z)
Supplement: Supplementary file 3 — Reporting Summary [file 41467_2023_38722_MOESM3_ESM.pdf]

nature portfolio

Corresponding author(s): Richard Benton

Last updated by author(s): Apr 18, 2023

Reporting Summary

Nature Portfolio wishes to improve the reproducibility of the work that we publish. This form provides structure for consistency and transparency in reporting. For further information on Nature Portfolio policies, see our Editorial Policies and the Editorial Policy Checklist.

Statistics

For all statistical analyses, confirm that the following items are present in the figure legend, table legend, main text, or Methods section.

n/a	Confirmed	
☐	☒	The exact sample size (*n*) for each experimental group/condition, given as a discrete number and unit of measurement
☐	☒	A statement on whether measurements were taken from distinct samples or whether the same sample was measured repeatedly
☐	☒	The statistical test(s) used AND whether they are one- or two-sided<br>*Only common tests should be described solely by name; describe more complex techniques in the Methods section.*
☒	☐	A description of all covariates tested
☐	☒	A description of any assumptions or corrections, such as tests of normality and adjustment for multiple comparisons
☐	☒	A full description of the statistical parameters including central tendency (e.g. means) or other basic estimates (e.g. regression coefficient) AND variation (e.g. standard deviation) or associated estimates of uncertainty (e.g. confidence intervals)
☐	☒	For null hypothesis testing, the test statistic (e.g. *F*, *t*, *r*) with confidence intervals, effect sizes, degrees of freedom and *P* value noted<br>*Give P values as exact values whenever suitable.*
☒	☐	For Bayesian analysis, information on the choice of priors and Markov chain Monte Carlo settings
☒	☐	For hierarchical and complex designs, identification of the appropriate level for tests and full reporting of outcomes
☒	☐	Estimates of effect sizes (e.g. Cohen's *d*, Pearson's *r*), indicating how they were calculated

Our web collection on statistics for biologists contains articles on many of the points above.

Software and code

Policy information about availability of computer code

Data collection	Locomotor activity monitoring was performed using the Drosophila activity monitor (DAM) system (PMID: 20972399) in incubators with continuous monitoring of light and temperature conditions (TriTech Research DT2-CIRC-TK).<br><br>High-speed imaging of oviposition behavior was performed with a high-speed camera (JAI RMC-6740 GE, IMACO as detailed in PMID: 31191270) using the custom FlyBehavior software (https://github.com/LMU-AgGompel/FlyBehaviour).<br><br>Electrophysiological recordings were performed with AutoSpike (Syntech).<br><br>Confocal imaging data were acquired with a Zeiss LSM 710 using ZEN 2.3 SP1 software.
Data analysis	Behavioral data were analyzed using Microsoft Excel; statistical analyses were performed in R.<br>Electrophysiological data were analyzed in AutoSpike and Microsoft Excel; statistical analyses were performed in R.<br>Confocal imaging data were processed with Fiji (PMID: 22743772).

For manuscripts utilizing custom algorithms or software that are central to the research but not yet described in published literature, software must be made available to editors and reviewers. We strongly encourage code deposition in a community repository (e.g. GitHub). See the Nature Portfolio guidelines for submitting code & software for further information.

Data

Policy information about availability of data

All manuscripts must include a data availability statement. This statement should provide the following information, where applicable:

- Accession codes, unique identifiers, or web links for publicly available datasets
- A description of any restrictions on data availability
- For clinical datasets or third party data, please ensure that the statement adheres to our policy

The datasets generated during this study are available in the Source Data files.

Human research participants

Policy information about studies involving human research participants and Sex and Gender in Research.

Reporting on sex and gender	Not applicable
Population characteristics	Not applicable
Recruitment	Not applicable
Ethics oversight	Not applicable

Note that full information on the approval of the study protocol must also be provided in the manuscript.

Field-specific reporting

Please select the one below that is the best fit for your research. If you are not sure, read the appropriate sections before making your selection.

[x] Life sciences [ ] Behavioural & social sciences [ ] Ecological, evolutionary & environmental sciences

For a reference copy of the document with all sections, see nature.com/documents/nr-reporting-summary-flat.pdf

Life sciences study design

All studies must disclose on these points even when the disclosure is negative.

Sample size	Preliminary analyses were performed to assess variance and determine the correct sample sizes and necessary conditions before conducting the experiments. Generally, there were 26-90 female flies in single-fly behavioral assays across 1-3 technical replicates, and 10-20 female flies (and an equivalent number of male flies) in group assays over 3 technical replicates. For electrophysiological recordings, 5-6 female flies were tested.
Data exclusions	In single-fly oviposition assays, flies laying fewer than two eggs were excluded for the calculation of the oviposition preference index.
Replication	All attempts of replication were successful. Additional replicates of certain experiments were made when used as controls or due to a higher variation in the data. In most oviposition assays, we ran experimental controls for each experiment, although due to space and time constraints they were not always run in parallel but performed during different days/weeks. All oviposition experiments were performed with similarly-aged flies under stringent temperature, humidity and light cycle conditions in an incubator, with the exception of the whole-fruit oviposition assays (Fig. 1b), which were run in a temperature- and humidity-controlled room under red light. The number of technical and biological replicates for each experiment is provided in the corresponding figure legend and/or Source Data files.
Randomization	For all experiments, we randomized the order of species, strains and genotypes, which generally depended upon the number of flies available for each experiment. Some genotypes were weaker and harder to culture, limiting the number of assays that we were able to perform in parallel. To control for potential variation in experimental conditions across days, we carefully collected similar sample sizes and performed the experiments and recorded the data at the same time (+/- 1 h).
Blinding	The investigators were not blinded during the course of the data collection and/or data analyses, as differences in the visual appearance of the animal culture medium and/oviposition substrate provided unavoidable information to the experimenter as to the conditions/genotypes being analyzed in many experiments. Analyses of behavioral experiments all relied on objective, quantitative criteria (e.g., egg number).

Reporting for specific materials, systems and methods

We require information from authors about some types of materials, experimental systems and methods used in many studies. Here, indicate whether each material, system or method listed is relevant to your study. If you are not sure if a list item applies to your research, read the appropriate section before selecting a response.

Materials & experimental systems

n/a	Involved in the study	
[ ]	[x]	Antibodies
[x]	[ ]	Eukaryotic cell lines
[x]	[ ]	Palaeontology and archaeology
[ ]	[x]	Animals and other organisms
[x]	[ ]	Clinical data
[x]	[ ]	Dual use research of concern

Methods

n/a	Involved in the study	
[x]	[ ]	ChIP-seq
[x]	[ ]	Flow cytometry
[x]	[ ]	MRI-based neuroimaging

Antibodies

Antibodies used	Guinea pig anti-Ir75b (RRID: AB_2631093); Alexa488 anti-guinea pig (A11073 INVITROGEN AG)
Validation	The Ir75b antibody was validated in previous publications (PMID: 28111079, 27776356 and 34362730) through its selective labeling of Ir75b neurons (identified independently using a transgenic marker) and loss of labeling in Ir75b mutant genotypes.

Animals and other research organisms

Policy information about studies involving animals; ARRIVE guidelines recommended for reporting animal research, and Sex and Gender in Research

Laboratory animals	All flies used for behavioral analyses were 5-6 day-old females. For group oviposition assays, 5-6 day-old males were also used. Details on all wild-type, mutant and transgenic genotypes used are provided in Supplementary Table 1.
Wild animals	This study did not involve wild animals.
Reporting on sex	Findings in this work only apply to female flies.
Field-collected samples	This study did not involve samples collected from the field.
Ethics oversight	All experiments were conducted in accordance with ethical guidelines from the University of Lausanne.

Note that full information on the approval of the study protocol must also be provided in the manuscript.
