# Supplementary material for: Odor-regulated oviposition behavior in an ecological specialist
Source: Nat Commun. 2023 May 26;14:3041. doi: 10.1038/s41467-023-38722-z (PMC10219952; doi:10.1038/s41467-023-38722-z)
Supplement: Supplementary file 4 — Description of Additional Supplementary Files [file 41467_2023_38722_MOESM4_ESM.pdf]

Description of Additional Supplementary Files:

Supplementary Movie 1. Compilation of video sequences illustrating substrate touching by the ovipositor in D. sechellia.

Supplementary Movie 2. Compilation of video sequences illustrating substrate scratching by the ovipositor in D. sechellia.

Supplementary Movie 3. Compilation of video sequences illustrating substrate digging by the ovipositor in D. sechellia.

Supplementary Movie 4. Compilation of video sequences illustrating indentation formation by the ovipositor in D. sechellia.

Supplementary Movie 5. Compilation of video sequences illustrating egg laying by D. sechellia.
